# Experimental Investigation of the Physical Properties and Microstructure of Slate under Wetting and Drying Cycles Using Micro-CT and Ultrasonic Wave Velocity Tests

**DOI:** 10.3390/s20174853

**Published:** 2020-08-27

**Authors:** Junwei Ma, Xiaoxu Niu, Chengren Xiong, Sha Lu, Ding Xia, Bocheng Zhang, Huiming Tang

**Affiliations:** 1 Three Gorges Research Center for Geo-Hazards of the Ministry of Education, China University of Geosciences, Wuhan 430074, China; majw@cug.edu.cn (J.M.); nxx@cug.edu.cn (X.N.); xiongcr@cug.edu.cn (C.X.); tanghm@cug.edu.cn (H.T.); 2 Faculty of Engineering, China University of Geosciences, Wuhan 430074, China; cug_xia@cug.edu.cn (D.X.); 20141002456@cug.edu.cn (B.Z.)

**Keywords:** micro-CT, ultrasonic wave velocity test, physical properties, microstructure, slate, wetting and drying cycles

## Abstract

Cyclic wetting and drying processes have been considered as important factors that accelerate the weathering process and have deteriorative effects on rock properties. In the present study, a fully nondestructive and noninvasive testing approach utilizing micro-CT and ultrasonic wave velocity tests was employed to investigate the microstructure of slate under wetting and drying cycles. We studied variations in the physical properties, including the dry weight and the velocities of P- and S-waves versus the number of wetting and drying cycles. The internal microstructural distributions were visualized and quantified by the 3D reconstruction and hybrid image segmentation of CT images. The degree of deterioration caused by wetting and drying cycles was reflected by exponential decreases of physical properties, including dry weight and velocities of the P- and S-waves. Parameters relating to the microfracture diameter, volume, etc. were quantified. The nondestructive and noninvasive testing approach utilizing micro-CT and ultrasonic wave velocity tests has potential for the detection and visualization of the internal microstructure of rock under wetting and drying cycles.

## 1. Introduction

Understanding and characterizing rock properties is crucial for the safety evaluation of rock slopes and landslides [1]. In practical engineering scenarios, rock slopes and landslides are substantially affected by environmental effects and human activities [2,3], thus accelerating rock weathering processes [4]. Among various environmental effects, the cyclic actions of wetting and drying caused by humidity changes have been considered important factors [2,5]. Cyclic wetting and drying phenomena resulting from seasonal changes in the groundwater level and variability in temperatures and rainfall [6] alter the rock properties, which over the year can eventually result in the movement and failure of rock slopes, as well as landslides [1,4]. Therefore, it is important to investigate the evolution of rock properties during cyclic wetting and drying processes.

Recently, the effects of cyclic wetting and drying processes on properties of various rocks (e.g., sandstone, mudstone, and argillaceous rocks [1,2,4,6,7]) have been well investigated by using various testing methods, such as scanning electron microscopy (SEM) [4,8], uniaxial compression testing [6,9,10], triaxial compression testing [11], creep testing [12], Brazilian tensile testing [13], and wave velocity testing [4]. The evolution characteristics under wetting and drying cycles have been investigated by studying changes in physical and mechanical properties, including the bulk density, porosity, weight loss, water absorption, wave velocity [4], elastic modulus, shear strength [14], tensile strength [15], uniaxial compression strength [10,16,17], and triaxial compression strength.

Although the effects of cyclic wetting and drying processes on rock properties have been intensively studied, these studies have mostly focused on strength, deformability, and failure modes, and limited works have investigated the internal microstructure. The degree of deterioration under wetting and drying cycles is not clearly understood. Moreover, most of the utilized testing methods are destructive.

The X-ray computed tomography (CT) technique was first developed in 1973 by Godfrey Hounsfield [18], who was later awarded the Nobel Prize in 1979. CT is a nondestructive and noninvasive technique that provides quantitative detection of an object’s interior structure based on the attenuation coefficient of the X-ray electromagnetic wave. It has been widely applied in various fields, such as medical tests [19], safety inspections, and material porosity detection [20,21]. Micro-CT, also called microtomography or microcomputed tomography [22], is a specialized type of CT on a small scale, with a greatly increased resolution ranging from (sub)micrometers to millimeters [23]. It has been extensively used for the quantitative investigation of the interior 3D microstructure of different rocks, including granites, sandstones, schists, metamorphic rocks, carbonate rocks, and shale [22,24,25,26,27,28,29,30,31,32,33,34,35]. However, studies are still insufficient in terms of investigating the interior microstructure of rock under wetting and drying cycles.

Slate is a typical metamorphic rock that is strongly characterized by foliation planes with weaker strength, and is widely distributed in the Qinling Mountain area in northwest China. It is frequently encountered in various engineering applications, such as dam foundation, tunneling, slope engineering, and hydrogeology [36,37,38]. Due to highly seasonal rainfall, the rock properties are substantially affected by wetting and drying cycles. It is expected that the effects of cyclic wetting and drying processes on slate characteristics are not comparable between sandstone, mudstone, and argillaceous rocks [38]. It is highly desirable to investigate the properties of slate under wetting and drying cycles.

The main objective of the present study was to investigate the deteriorative effects of wetting and drying cycles on the physical properties and internal microstructure of slate. A nondestructive and noninvasive approach utilizing micro-CT and ultrasonic wave velocity tests was employed. The physical properties of slate subjected to different wetting and drying cycles were tested. The evolution characteristics of the physical properties under wetting and drying cycles were investigated. The internal microstructural distributions were visualized and quantified by a 3D reconstruction and hybrid image segmentation approach.

## 2. Materials and Methods

### 2.1. Micro-CT Test System

In the present study, a Phoenix V|tome|x S micro-CT system (GE Sensing & Inspection Technologies, Cincinnati, OH, USA) was employed for rock specimen scanning. This system is composed of an X-ray generator, a detector, a translation system, and a computer system for motion control and data acquisition. The samples used for scanning attenuate the passage of X-ray radiation as a function of the material composition, density, and thickness along the beam direction [22]. The Phoenix V|tome|x S micro-CT system is equipped with a 240 kV/320 W microfocus X-ray tube. More features of the Phoenix V|tome|x S micro-CT system are listed in Table 1.

### 2.2. Ultrasonic Wave Velocity Test

Ultrasonic wave velocity tests are a nondestructive technique for the measurement of the velocities of P- and S-waves. The velocities of P- and S-waves traveling in a rock sample are highly related to the density and elastic properties of the rock. Therefore, the ultrasonic wave velocity test has been widely used for rock property testing, and the velocities of P- and S-waves have been widely used as the most important indices of rock properties [39].

In the present study, a SonicViewer-SX (model-5251C) with a 200 kHz P-wave transducer and 100 kHz S-wave transducer (OYO Corporation, Japan) was employed for the ultrasonic wave velocity test. More features of the SonicViewer-SX (model-5251C) are listed in Table 1.

### 2.3. Preparation of the Rock Specimens and Experimental Procedure

**Figure 1:**
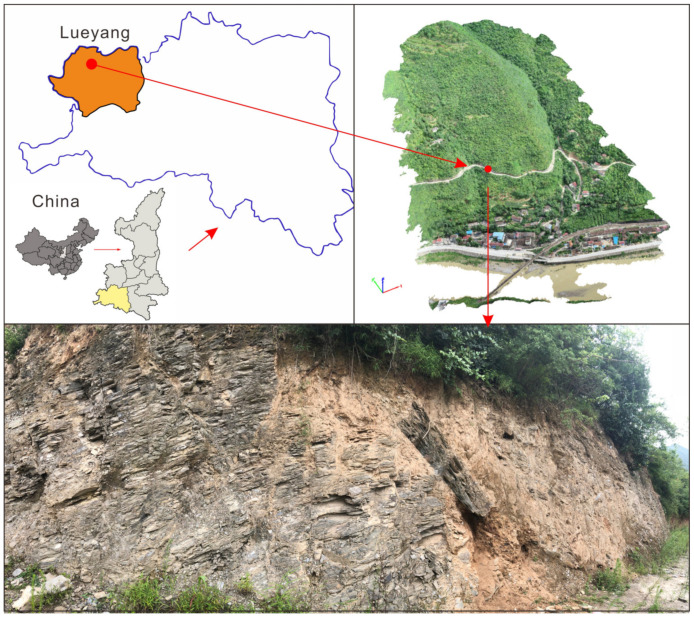
Study site and sampling position.

**Figure 2:**
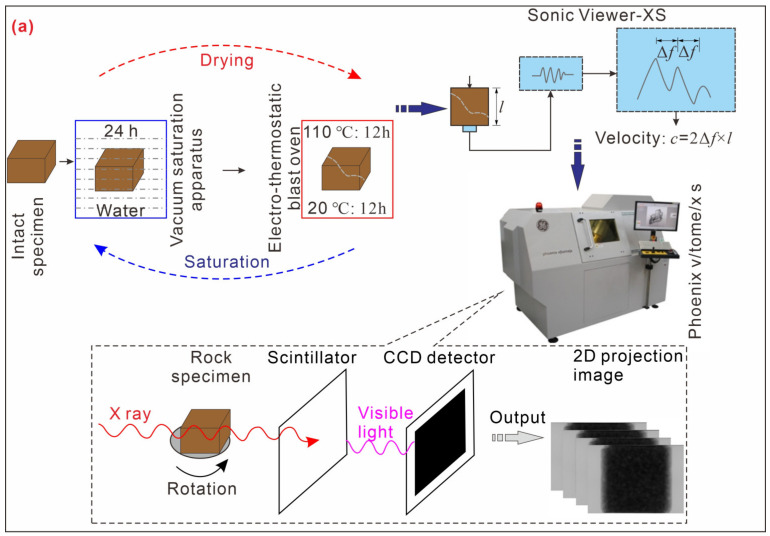

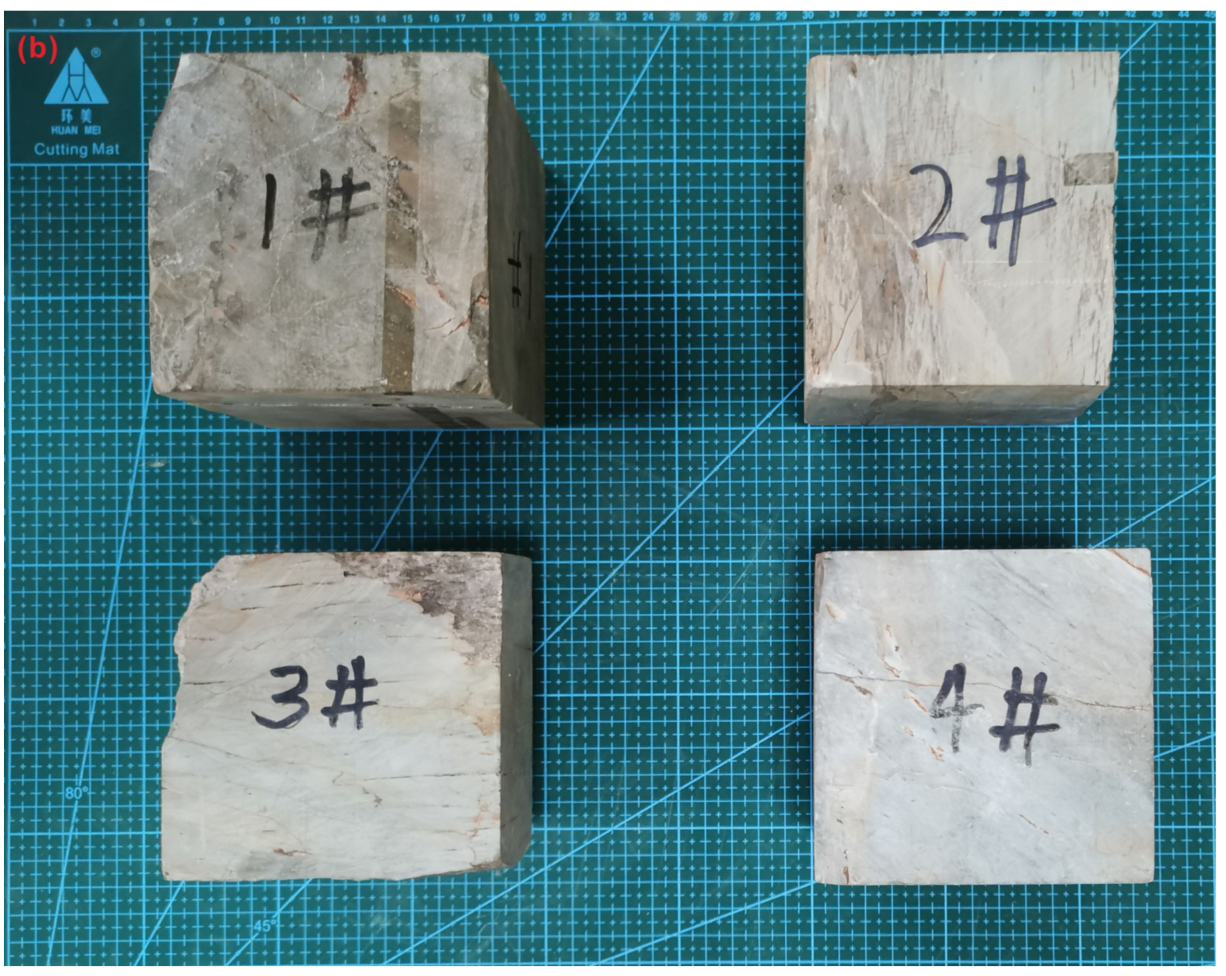
(**a**) Schematic of the experimental process of the cyclic wetting and drying treatment. (**b**) Slate specimens after six cycles of the wetting and drying treatment.

The intact slate samples used for the wetting and drying experiments were collected from Lueyang County, Qinling Mountain area in Shaanxi Province, China (latitude and longitude coordinates: E 106°4′1.66″, N 33°34′39.8″, Figure 1). The slate is composed of minerals including quartz, muscovite, albite, chlorite, and illite. The average dry density and moisture contents are 2.45 g/cm^3^ and 2.83%, respectively. The raw slate samples were cut in the form of cubes with dimensions of 100 mm × 100 mm × 100 mm. Four intact specimens were chosen to investigate the microstructure under wetting and drying cycles. The wetting and drying treatment mainly consisted of two processes: saturation (from a dry state to a saturated state) and drying (from a saturated state to a dry state). In each saturation process, the specimens were submerged into distilled water for saturation for 24 h in a vacuum saturation apparatus. In each drying process, the saturated specimens were placed in an electro-thermostatic blast oven at 110 °C for 12 h and dried to a constant weight at 20 °C for 12 h (schematically illustrated in Figure 2a). In the present study, intact slate specimens without cyclic wetting and drying treatments were considered to undergo 0 wetting and drying cycles (*n* = 0). Six cycles of wetting and drying treatment (*n* = 6) were performed for each specimen. Figure 2b shows the rock samples after six cycles of wetting and drying treatments. The physical properties, including the dry weight and wave velocity, under each wetting and drying cycle (from 0 to 6) were tested (schematically illustrated in Figure 2a). After six cycles of the wetting and drying treatment, micro-CT scans were performed (schematically illustrated in Figure 2a) at the Faculty of Engineering, China University of Geosciences. The voltage and current of the X-ray tube for micro-CT scanning were set to 200 kV and 190 μA, respectively. A total of 1800 two-dimensional projection images with 2000 × 2000 pixels were obtained for each slate specimen. After scanning and reorientation of the samples, images with a voxel size of 80 μm × 80 μm × 80 μm were produced. More details of the parameter values of the micro-CT scanning configuration are listed in Table 2.

### 2.4. CT Image Processing

**Figure 3:**
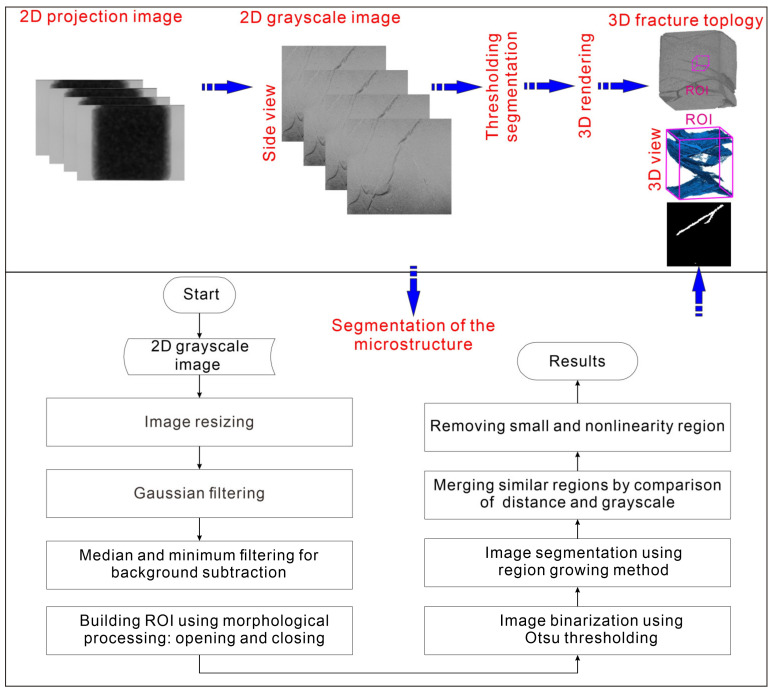
The workflow of the CT imaging analysis procedure.

In the present study, raw projection images were processed by 3D reconstruction and hybrid image segmentation (schematically illustrated in Figure 3) in order to quantitatively analyze the internal microstructure. The 3D reconstruction of raw projection images and quantitative analysis were performed with the GE phoenix datos|x 2.5.2 software (GE Sensing and Inspection Technologies, Wunstorf, Germany) and VGSTUDIO MAX 3.3.0, respectively (Volume Graphics GmbH, Heidelberg, Germany). This process was based on practical experiences in the literature [40,41,42,43,44]. Thresholds for the segmentation were carefully set by observation of the grayscale histogram distribution of microstructure and rock fragments. After 3D rendering, a region of interest (ROI) was selected by the particle analysis tool in VGSTUDIO MAX for estimation of the microstructure. Characteristics including the diameter, volume, and area were obtained. Moreover, the microstructures were segmented through hybrid image segmentation following the processes of image resizing, Gaussian filtering, median and minimum filtering for background subtraction, building ROI using morphological processing of opening and closing operations, image binarization using Otsu thresholding, image segmentation using region growing method, merging similar regions by comparison of distance and grayscale, and removing small and nonlinearity region (Figure 3). The parameter settings for segmentation of the microstructure are listed in Table 3.

## 3. Results

### 3.1. Physical Properties Subjected to Different Wetting and Drying Cycles

**Figure 4:**
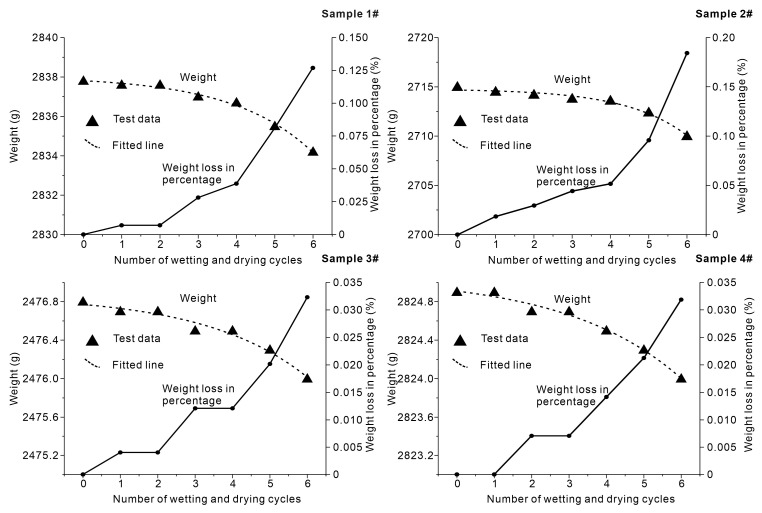
Variation in the sample weight versus the number of wetting and drying cycles.

The dry weight of slate specimens under wetting and drying cycles is shown in Figure 4. The dry weight of slate specimens decreased with the increase in the number of wetting and drying cycles. After six cycles of wetting and drying treatments (*n* = 6), the sample dry weight was reduced by 3.6, 5, 0.8, and 0.9 g for the four samples. The widely used empirical exponential equation for describing rock characterization and damage [4,45,46,47] was chosen for experimental data fitting. The results show that the relationship between sample weight and the number of wetting and drying cycles could be described satisfactorily by an exponential equation. The best-fit lines are shown in Figure 4. The obtained equations are listed in Table 4. The obtained results show that the exponential equations were equally effective, with coefficients of determination of 0.99, 0.98, 0.97, and 0.98 for the four samples.

**Figure 5:**
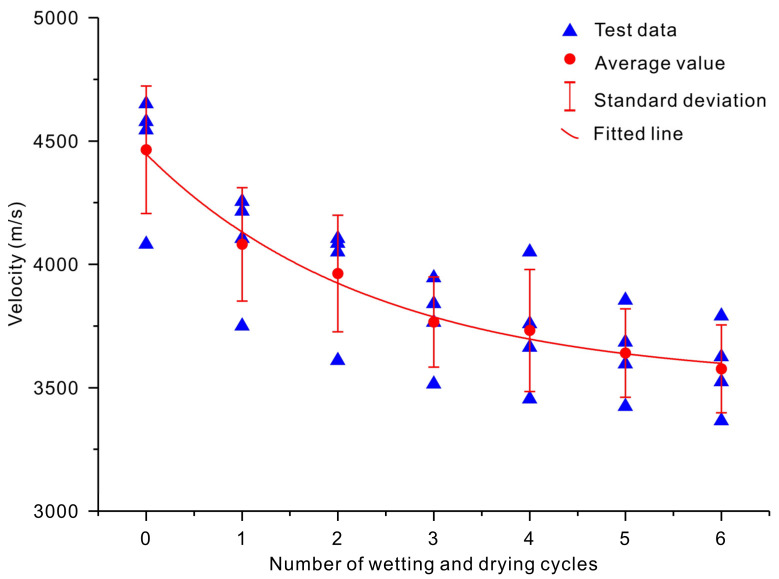
Variation in the P-wave velocity versus the number of wetting and drying cycles.

**Figure 6:**
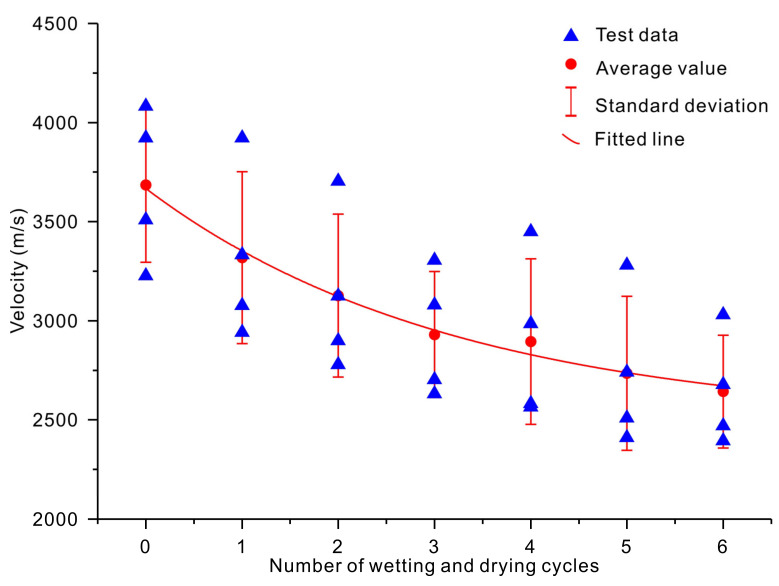
Variation in the S-wave velocity versus the number of wetting and drying cycles.

Figure 5 and Figure 6 show the variations in the velocities of P- and S-waves versus the number of wetting and drying cycles, respectively. Both the P- and S-wave velocities decreased with an increase in the number of wetting and drying cycles (Figure 5 and Figure 6). The initial (*n* = 0) P-wave velocities of the slate specimens were 4545, 4082, 4651, and 4578 m/s for the four samples. The initial (*n* = 0) S-wave velocities of the slate specimens were 3509, 3226, 4082, and 3922 m/s. After being subjected to six cycles of wetting and drying treatment (*n* = 6), the P-wave velocities were reduced by 20.24%, 17.56%, 18.51%, and 23.02%, respectively (Figure 5). The S-wave velocities were reduced by 29.61%, 25.79%, 25.77%, and 31.72%, respectively (Figure 6). Empirical exponential equations were applied to describe the relationship between the velocities of P- and S-waves and the number of wetting and drying cycles. The best-fit lines and obtained exponential equations are shown in Figure 5 and Figure 6 and listed in Table 4. The obtained results show that exponential regression satisfactorily presented the relationship between the velocities of P- and S-waves and the number of wetting and drying cycles, with coefficients of determination of 0.99 and 0.99, respectively.

Based on previous studies [8,48], we speculate that the decreasing trends for velocities of P- and S-waves could be explained by the increase in porosity and the degradation of rock materials caused by the combined effects of physical and chemical damage [1,49,50]. First, internal water within rock specimens has combined effects, including dissolution, erosion, and softening effects on rock minerals. Under these combined effects, the cementation between mineral grains deteriorates and does not recover, even though the saturated rock specimen is dried. Thus, the velocities of P- and S-waves are characteristic of an attenuation trend. Second, the water absorption and desorption of swelling clay minerals (e.g., smectite, illite, and kaolinite) within rock specimens would cause the loading and unloading of tensile stresses, accelerating the growth and propagation of internal microcracks. Therefore, with the increase in the number of wetting and drying cycles, the number of internal microfractures within the rock specimen increase. The increase in microfractures has a deteriorative effect on rock properties such as wave velocities mechanical properties.

### 3.2. Visualization of Microfracture Clusters

**Figure 7:**
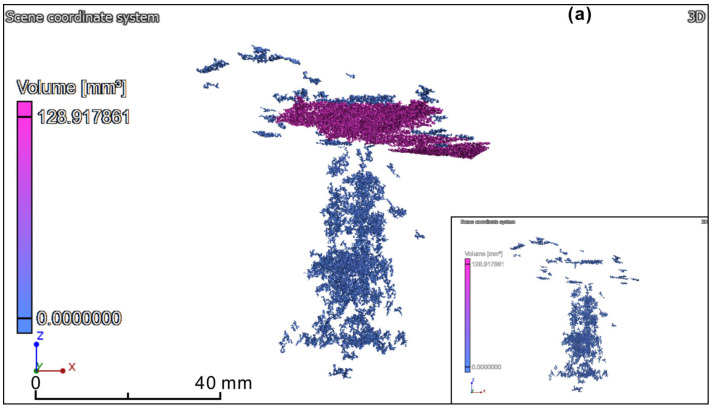

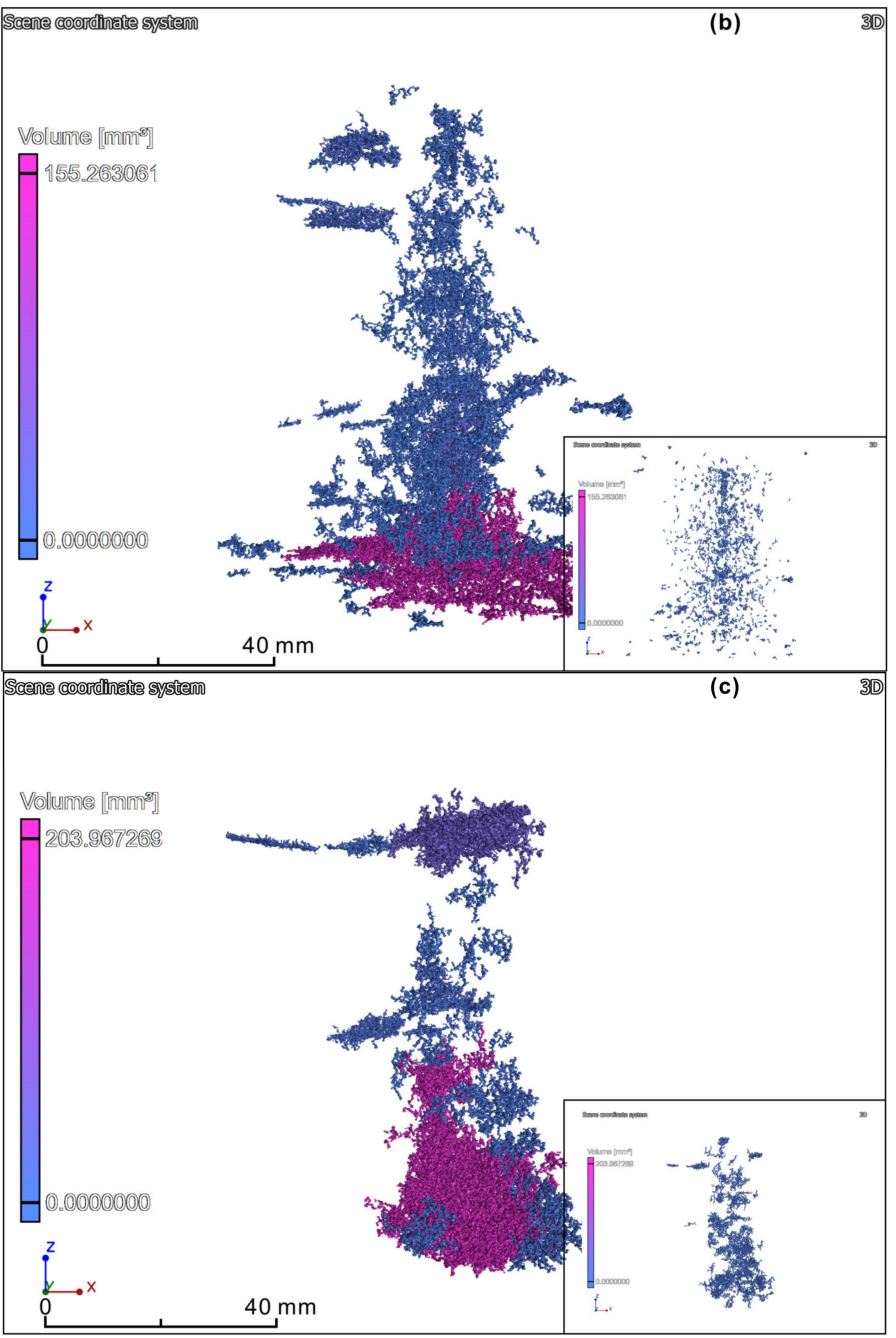

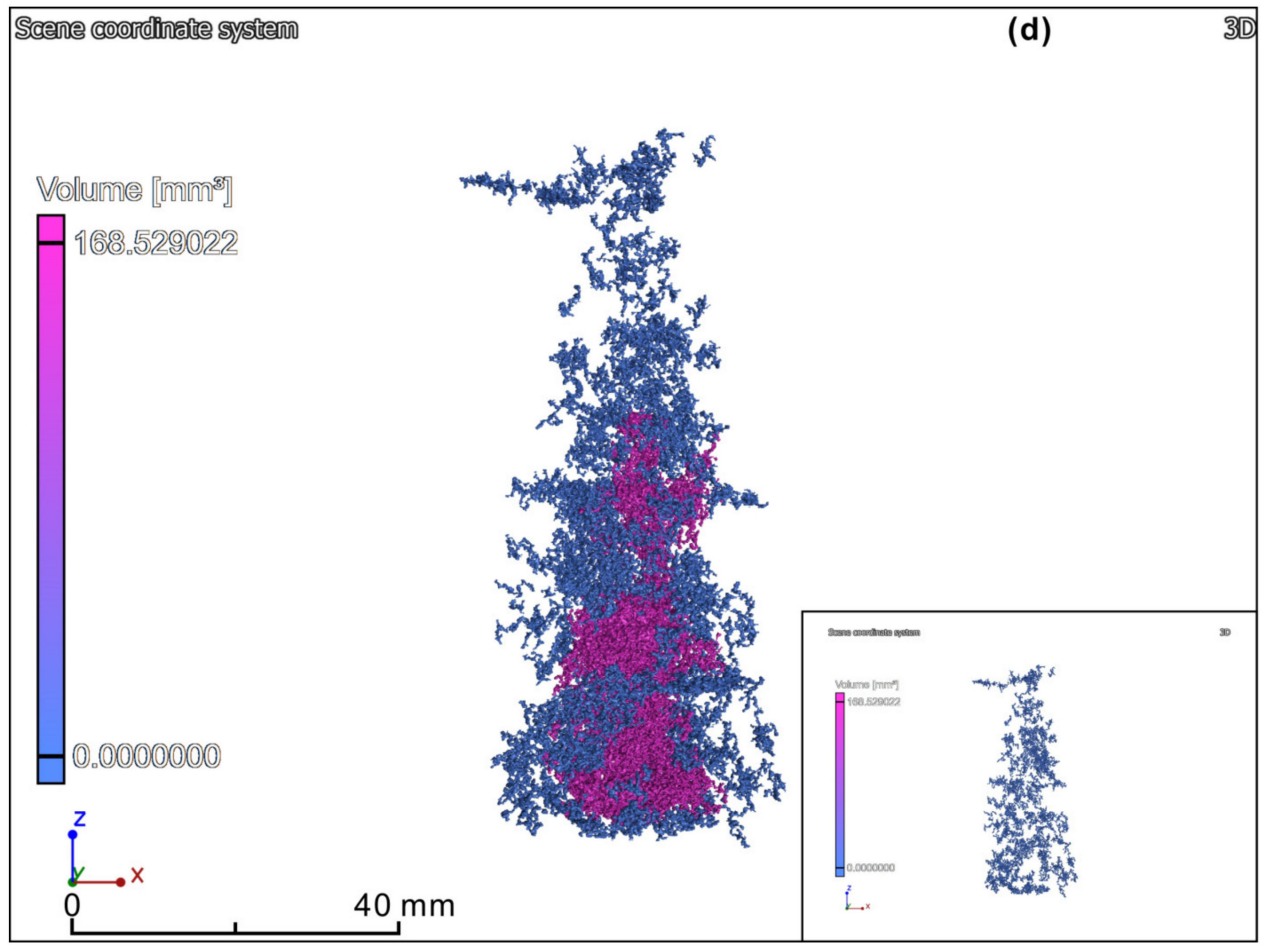
Reconstructed 3D distribution of microfracture clusters within sample specimens before (shown in insets) and after wetting and drying treatments. (**a**) Sample 1; (**b**) Sample 2; (**c**) Sample 3; (**d**) Sample 4.

The 3D distributions of microfractures within the specimens before and after wetting and drying treatments were reconstructed and visualized using gradient colormaps, which facilitate visualization of the complexity and interconnectivity of the microfracture clusters. As shown in Figure 7, before wetting and drying treatments, the microfractures within the specimens were not well developed, and only poorly connected microfractures could be observed. The defect microfracture volumes were 276.84, 427.82, 369.79, and 351.62 mm^3^, respectively (Table 5). The defect volume ratios were 0.12%, 0.09%, 0.12%, and 0.09%, respectively (Table 5).

A comparison of the images before wetting and drying treatments shows clearly that the latter contained more microfractures than the former. After subjected to six cycles of wetting and drying treatment (*n* = 6), the microfracture volumes and maximum pore equivalent diameter were significantly increased. The defect volume ratios significantly reached 0.22%, 0.34%, 0.30%, and 0.28% in the four samples. For example, one set of partially connected microfractures developed in the x–y plane within sample 1; the maximum pore equivalent diameter was 49.55 mm. The microfractures were mainly distributed in the x–y plane and oriented perpendicular to the direction of foliation planes. This can be explained as follows: due to the processes of metamorphism under high temperature and high pressure, slate is strongly characterized by foliation planes with weaker strength. The foliation planes are more prone to wetting and drying treatments. Therefore, the microfractures were mainly generated in the preferred direction along the foliation planes. This finding is consistent with previous observations from scanning electron microscopy images [33,51], which revealed that microcracks were generally controlled by the bulk rock mineral geometry.

**Figure 8:**
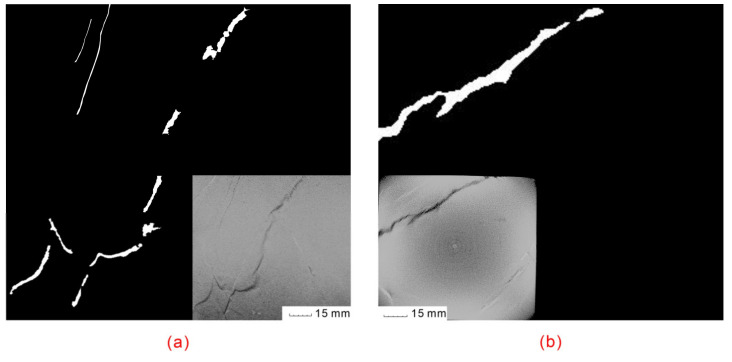
Results of microfracture segmentation and the original CT (shown in insets) after six wetting and drying cycles for sample 1. (**a**) At the center (z = 0 mm) of the scene coordinate system. (**b**) At z = 10 mm of the scene coordinate system.

To better visualize the interconnectivity of the microfractures after six wetting and drying cycles, fractures were segmented from the original CT image for sample 1. The result of the fracture segmentation is shown in Figure 8. The experimental results demonstrate that the proposed approach can segment the microfracture effectively. As shown, a partially connected microfracture was formed throughout sample 1. The connectivity length was at the centimeter scale.

## 4. Conclusions

This study, to the best of our knowledge, is the first attempt to investigate the physical properties and microstructure of slate under wetting and drying cycles using a fully nondestructive and noninvasive approach utilizing micro-CT and ultrasonic wave velocity tests. The following conclusions can be drawn:

Decreases of physical properties reflected the degree of deterioration. The experimental results show that with an increasing number of cycles, the specimen dry weight and velocities of the P- and S-waves decreased. Empirical exponential equations could satisfactorily represent those relationships, with coefficients of determination close to one.

3D reconstruction and the hybrid image segmentation approach provide a visual representation and quantitative analysis of the macropore distribution. Parameters relating to the microfracture diameter, volume, etc. were quantified.

Given the successful use of micro-CT and ultrasonic wave velocity tests, this nondestructive and noninvasive testing approach may be a promising method for detecting and visualizing the internal microstructure of rock samples under wetting and drying cycles.

## Figures and Tables

**Table 1:** Main features of the Phoenix V|tome|x S and SonicViewer-SX (model-5251C).

Experimental Equipment	Specifications
Phoenix V|tome|x S	X-ray tube type: Open directional high-power microfocus X-ray tube, with a closed cooling water circuit
	Max. voltage/power: 240 kV/320 W
	Geometrical magnification (3D): 1.46× to 100× with a microfocus tube
	Detail detectability: Down to <1 µm (microfocus tube);
	Max. focus object distance: 545 mm (microfocus tube)
SonicViewer-SX (model-5251C)	Output voltage: 500 V
	Pulse width: 6 μs ± 2 μs
	Input impedance: 1 MΩ
	Gain: 1, 2, 5, 10, 20, 50, 100, 200
	Frequency range: 10–1000 kHz
	Low-pass filter: 200 kHz
	A/D resolution: 12 bit
	Sample rate: 50, 100, 200, 500, 1000, 2000 ns
	Data length: 1024
	P-wave transducer: 200 kHz
	S-wave transducer: 100 kHz

**Table 2:** Micro-CT scanning configuration parameters.

Parameters	Value
Magnetization	2.50
Source to sample distance (FOD, mm)	372.57
Source to detector distance (FDD, mm)	931.43
Number of projections	1800
Image width (pixel)	2000
Image height (pixel)	2000
Detector type	DXR-250
Acquisition time (ms)	1000.10
Camera binning	1
X-ray voltage (kV)	200
X-ray current (μA)	190
Voxel size (μm × μm × μm)	80 × 80 × 80

**Table 3:** Parameter settings for the segmentation of the microstructure.

Process	Parameter	Process	Parameter
Image resizing	256 × 256 pixels	Gaussian filtering	10 × 10 pixels
Median filtering	10 × 10 pixels	Minimum filtering	10 × 10 pixels
Morphological opening	Disk, R = 3 pixels	Morphological closing	Disk, R = 3 pixels
Region growing	Normalized distance < 0.1	Merging similar regions	Distance < 10 pixels and mean grayscale < 40
Removing small regions	<200 pixels	Removing nonlinearity regions	Major axis length/Minor axis length < 3

**Table 4:** Relationships between the number of wetting and drying cycles and rock properties (*n*: number of wetting and drying cycles).

Measurement of Properties	Relationship Equations	Coefficient of Determination (R^2^)
Dry weight (*w*)	Sample 1#: *w*_1_ = 2838.02754 − 0.21754 × e^0.47957n^	0.99
	Sample 2#: *w*_2_ = 2714.78392 − 0.1083 × e^0.62916n^	0.98
	Sample 3#: *w*_3_ = 2476.86398 − 0.09339 × e^0.36783n^	0.97
	Sample 4#: *w*_4_ = 2825.05996 − 0.15042 × e^0.32507n^	0.99
P-wave velocity (*v_P_*)	*v_P_* = 3523.69446 + 923.46222 × e^−0.41864n^	0.99
S-wave velocity (*v_S_*)	*v_S_* = 2488.97977 + 1178.33291 × e^−0.31083n^	0.99

**Table 5:** Measurement of the microfracture clusters obtained from CT scans for the four samples before (*n* = 0) and after (*n* = 6) wetting and drying treatments.

		Sample 1	Sample 2	Sample 3	Sample 4
ROI dimensions (mm × mm × mm)	*n* = 0	50 × 50 × 50			
	*n* = 6				
Maximum pore equivalent diameter (mm)	*n* = 0	9.85	4.37	9.21	4.99
	*n* = 6	49.55	41.39	27.65	31.34
Average diameter (mm)	*n* = 0	4.16	1.44	5.13	3.56
	*n* = 6	4.43	4.35	9.28	4.05
Defect volume of microfractures (mm^3^)	*n* = 0	147.92	116.51	155.11	115.42
	*n* = 6	276.84	427.82	369.79	351.62
Total area of microfractures (mm^2^)	*n* = 0	4084.23	3090.52	4311.45	375.40
	*n* = 6	6880.27	11,258.71	9905.19	9664.15
Defect volume ratio (%)	*n* = 0	0.12	0.09	0.12	0.09
	*n* = 6	0.22	0.34	0.30	0.28
